# Supplementary material for: Single-cell lipidomics enabled by dual-polarity ionization and ion mobility-mass spectrometry imaging
Source: Nat Commun. 2023 Aug 25;14:5185. doi: 10.1038/s41467-023-40512-6 (PMC10457347; doi:10.1038/s41467-023-40512-6)
Supplement: Supplementary file 1 — Supplementary Information [file 41467_2023_40512_MOESM1_ESM.docx]

***Supplementary Information for***

Single-cell lipidomics enabled by dual-polarity ionization and ion mobility-mass spectrometry imaging

Hua Zhang^1^, Yuan Liu^1^, Lauren Fields^2^, Xudong Shi^3^, Penghsuan Huang^2^, Haiyan Lu^1^, Andrew J. Schneider^4^, Xindi Tang^2^, Luigi Puglielli^4, 5^, Nathan V. Welham^2^, and Lingjun Li^1,2,6,7,*^

^1^School of Pharmacy, University of Wisconsin-Madison, Madison, Wisconsin 53705, USA

^2^Department of Chemistry, University of Wisconsin-Madison, Madison, Wisconsin 53706, USA

^3^Division of Otolaryngology, Department of Surgery, School of Medicine and Public Health, University of Wisconsin-Madison, Madison, Wisconsin 53792, USA

^4^Department of Medicine, University of Wisconsin-Madison, Madison, WI 53705, USA

^5^Waisman Center, University of Wisconsin-Madison, Madison, WI 53705, USA

^6^Lachman Institute for Pharmaceutical Development, School of Pharmacy, University of Wisconsin-Madison, Madison, WI 53705, USA

^7^Wisconsin Center for NanoBioSystems, School of Pharmacy, University of Wisconsin-Madison, Madison, WI 53705, USA

| **Corresponding author:** | Lingjun Li |
| --- | --- |
| **Post address:** | 777 Highland Ave, Madison, Wisconsin, United States |
| **ZIP Code:** | 53705 |
| **Email:**  **Tel:**  **Fax:** | lingjun.li@wisc.edu  +1 608-265-8491  +1 608-262-5345 |

**Supplementary Methods**

**Chemicals and materials**

Chemicals such as methanol (MeOH), ethanol (EtOH), acetonitrile (ACN), 2-propanol (IPA), peracetic acid (30% in acetic acid, w/w), distilled water, trichloromethane, ammonia methanol (2 M) were all optima grade and purchased form Fisher Scientific (Pittsburgh, PA). DMSO and stearoyl-CoA desaturase inhibitor of MF-438 were purchased from Millipore Sigma. Indium tin oxide (ITO)-coated glass microscope slides (25 mm × 75 mm × 1 mm) were purchased from Bruker (Billerica, MA, United States).

**Drug treatment of cells**

MF-438, a stearoyl-CoA desaturase inhibitor[^1^](#_ENREF_1), was dissolved in DMSO at 50 mM and stored at –80 °C until use. PSC cells were seeded in cell-culture treated opaque 96 well plate (1000/well) in triplicate and allowed to attach overnight. After attachment, the medium was replaced by serum free medium, and cells were treated with MF-438 of different concentrations (0, 1, 10, 100, 1000, 10000, 20000 pM). Cell viability was measured by CellTiter-Glo® 2.0 (Promega) analysis after 48 hours treatment. Luminescence was measured using microplate reader. The cell viability assay indicates that the MF-438 drug has significant effects on the PSC cell proliferation with concentration over 10 nM. To study the effects of MF-438 on PSCs, cells were seeded on ITO slides with serum free culture medium. After incubation overnight, the drug treatment group was treated with 10 nM MF-438 and the control group was spiked with the vehicle of DMSO. After 48 hours treatment, PSCs on ITO slides were used for MALDI MSI experiments.

**HPLC-NanoESI-MS/MS of cellular/brain lipid extracts on Orbitrap platform**

For conventional lipidomics analysis, cellular lipid extracts were subjected to high performance liquid chromatography (HPLC)-NanoESI-MS/MS interrogation. The extraction of cellular lipids was based on a modified Folch protocol. Briefly, cell suspensions (ca. 2 × 10^6^ cells) were centrifuged at 400 xg for 2 min to obtain the cell pellets, and the cells were washed with 1 mL of cold dPBS for three times (400 g and 2-min centrifugation). For lipid extraction, 0.5 mL of water, 0.5 mL of methanol, and 0.5 mL of chloroform were added into the tube. The mixture was vigorously vortexing for 2 min and then sonicated in a water bath for 15 min following by centrifugation (12000 xg) at 4 °C for 10 min. After the centrifugation, the chloroform layer was transferred to a screw-capped glass tube. The extraction was repeated once, and the chloroform layers were combined and dried under a nitrogen stream. All the lipid extracts were stored at −80 °C before analysis.

The HPLC-nanoESI-MS analysis was carried out on Orbitrap Fusion™ Lumos™ Tribrid™ mass spectrometer equipped with a Dionex UltiMate 3000 UPLC system (Thermo Scientific, San Jose, CA, USA). Liquid chromatography was performed using a homemade microcapillary column (ID of 75 μm) which was packed with 14 cm of Bridged Ethylene Hybrid C18 particles (1.7 μm, 130 Å, Waters), and the microcapillary column was fabricated as the nanoESI emitter tip. Mobile phase A is composed of ACN/water (40/60, v/v) and mobile phase B is IPA/ACN (90/10, v/v), both the A and B phases contain 1.0 mM ammonium acetate to help the ionization of lipids. Chromatographic separation was performed using a 115-min gradient at a flow rate of 200 nL min^-1^: 0-4 min, 5% B; 4-5 min, 5-30% B; 5-10 min, 30-35% B; 10-15 min, 35-51% B; 15-40 min, 51-61% B; 40-50 min, 61-70% B; 50-60 min, 70-99% B; 60-75 min, 99% B; 75-76 min, 99-60% B; 76-79 min, 60% B; 79-80 min, 60-99% B; 80-85 min, 99% B; 85-90 min, 99-5% B; 90-115 min, 5% B. The cellular lipid extracts were reconstituted in 100 *μ*L IPA/ACN/water solution (v/v/v, 45/25/30) for HPLC-MS/MS analysis. Mass spectra were collected over a mass range of *m/z* 200–1200 in both positive and negative ion detection modes. The ionization voltage was set to ±2.0 kV and the heated ion transportation capillary was maintained at 320 °C. Survey scans of lipid precursor ions were performed at a resolving power of 60k (at *m/z* 200) with an AGC target of 1 × 10^5^ and maximum injection time of 100 ms. For MS/MS, mass spectra were collected over a mass range of *m/z* 100–1200, high-energy collisional dissociation (HCD) was performed with precursor ion isolation window width of 0.8 Da, and normalized collision energy (NCE) was set to 25%. Other MS instrumental parameters were set to default values without any further optimization.

**Cellular lipid extract analysis via UPLC-nanoESI-MS/MS on timsTOF fleX instrument**

The UPLC-nanoESI-MS/MS analysis was carried out on timsTOF fleX mass spectrometer. (Bruker Daltonics, Bremen, Germany) equipped with a Waters Acquity M-Class ultra-high pressure nanoflow chromatography system. Lipids were separated with a binary gradient at a constant flow rate of 200 nL/min on a home-built reversed-phase microcapillary column (14 cm x 75 μm i.d.), which was packed with Bridged Ethylene Hybrid C18 particles (1.7 μm, 130 Å, Waters). Mobile phases A and B were ACN/water (40/60, v/v) and IPA/ACN (90/10, v/v), both contain 10 mM ammonium formate. The 115 LC-MS gradient was set as: 0-0.1 min, 5%B; 0.1-63.5 min, 5-90%B; 63.5-64 min, 90-95%B; 64-80 min, 95%B; 80-90 min, 95-5%B; 90-115 min, 5%B. The cellular lipid extracts were reconstituted in 100 μL IPA/ACN/water solution (v/v/v, 45/25/30) for UPLC-nanoESI-MS/MS analysis.

The nanoLC was coupled to a hybrid trapped ion mobility-quadrupole time-of-flight mass spectrometer (timsTOF fleX MALDI-2, Bruker Daltonics, Bremen, Germany) via a modified nano-electrospray ion source (Captive Spray, Bruker Daltonics). The accumulation and ramp time were set at 100 ms in positive ion mode and 300 ms in negative ion mode for the TIMS tunnel. The mass spectra were recorded in the range from *m/z* 100-1350 in both positive and negative electrospray modes. The ion-mobility energy ramping was set from 0.6 to 1.95 Vs/cm^2^. Precursors for data-dependent acquisition were fragmented with an ion mobility-dependent collision energy, which was set linearly from 25 to 55 eV in both ion modes. The total acquisition cycle of 0.32 s includes one full TIMS-MS scan and two PASEF MS/MS scans, which the precursor ions with an intensity above the threshold of 100 counts and below the target value of 4000 counts were repeatedly scheduled for PASEF MS/MS scans. Other ions were dynamically excluded for 0.1 min. The TIMS ion charge control was set to 7.5e6.

The raw data files were analyzed with MetaboScape version 8.0.1 (Bruker Daltonics, Germany). Lipid annotations of detected ion features were performed with “Lipid Species Annotation” function and searched with spectral libraries (MSDIAL-TandemMassSpectralAtlas-VS68-Pos&Neg). Lipid identifications were further filtered with delta *m/z* between -10 to 10 ppm, delta CCS values between -5 to 5%, and MS/MS spectra matching to spectral libraries.

**Evaluation of reproducibility and accuracy of SC-MALDI-MSI**

Lipid standards were performed to evaluate the reproducibility and accuracy of SC-MALDI-MSI. Briefly, the blank ITO slide was pre-coated with DAN matrix via sublimation, then, the lipid standards of LPC 18:1 and PC (18:1-18:1) at a concentration of 0.1 µg mL^−1^ in 70% ACN were sprayed onto the surface of the matrix. Thus, the lipids standards were relatively homogeneous deposited on the ITO slide. The working parameters of the M5-Sprayer are as following: a flow rate of 20 μL min^−1^, tracking space of 2 mm, 8 passes, nozzle temperature of 60 °C, nozzle nitrogen gas pressure of 10 psi, and moving velocity of the nozzle was 1000 mm min^−1^, drying time between each pass was set to 30 s.

Then the lipid standard slide was subjected to MALDI-MSI analysis under the identical parameters of single-cell MS imaging, in which 12 separate square regions (ca. 200 µm × 200 µm, 400 pixels of each) on the lipid standard slide were selected for MS imaging. A box plot of the signal intensities of LPC 18:1 ([M+H]^+^, *m/z* 522.3545) and PC (18:1-18:1) ([M+H]^+^, *m/z* 786.6007) from each spectrum/pixel were shown in **Supplementary Figure 6**. Among the 12 regions, the relative standard deviations (RSDs) were 9.5%–10.5% for the signal intensities of LPC 18:1 and the RSDs were 11.8%–14.3% for PC (18:1-18:1), while the average intensities of LPC 18:1 and PC (18:1-18:1) among the 12 regions were quite consistent, with RSDs (n = 12) of 1.35% and 1.67% for LPC 18:1 and PC (18:1-18:1), respectively. Also, the average mass spectra from the 12 regions show the peaks of LPC 18:1 and PC (18:1-18:1) have a good alignment between different regions as no mass shift was found. The results indicate that the SC-MALDI-MSI could offer good reproducibility and mass accuracy for single-cell analysis. Indeed, signal intensity variations were found within each region, this might be largely due to the fact that the deposition of lipid standards was not fully homogeneous using a pneumatic sprayer and the lipid solution microdroplets could affect the MALDI matrix crystal upon its landing on the surface.

**Evaluation of single-cell washing and fixing pretreatments in SC-MALDI-MSI**

Cell sample washing and fixing are commonly applied procedures in single-cell MS imaging (e.g., Anal. Chem. 93, 4513–4520 (2021); Nature Methods 18, 799–805 (2021)). Similar as the previous studies, the cells cultured on the ITO slides went thorough washing or fixing steps including phosphate buffer saline (1x PBS) washes (clean the residual culture media), fixing with chilled 4% formaldehyde PBS for 15 min, and 50 mM ammonium acetate aqueous washes. The cultured cells were first washed using PBS to clean the residual medium. Using PBS to wash cells is a standard practice in cell culture and is generally considered to have minimal impact on the cell lipidome. As we know that, PBS is an isotonic solution that does not cause osmotic shock to the cells and is commonly used for a variety of cell culture applications, such as washing cells before dissociation, transporting cells or tissue samples, and diluting cells for counting.

To evaluate the sample washing and fixing pretreatments in SC-MALDI-MSI, samples of a lipid standard mixture and the cellular lipid extracts from the ITO slide were prepared for the validation experiments. The lipid standard mixture sample includes FA 16:1, FA 18:1, LPC 18:1, PC 18:1-18:1, PC 18:0-18:1, PS 18:0-18:1, PE 18:0-18:1 at 1 ug/mL of each. We used the lipid standard mixture solution as a simulated sample, the sample was treated with 4% formaldehyde PBS and 50 mM ammonium acetate, then the lipids were extracted via Folch's extraction procedure, and subjected to nanoESI analysis using the Orbitrap Fusion Lumos mass spectrometer. As a control experiment, the sample was treated with deionized (DI) water instead of ammonium acetate and formaldehyde PBS. The result of the lipid standard mixture experiments is shown in **Supplementary Figure 25**, showing that the lipid signals are quite consistent between the washing/fixing treated and the control sample. This result indicates that the washing and fixing pretreatments applied prior to SC-MSI have minimal impact on the tested lipid standard mixture sample.

Additionally, we extracted cellular lipids from the ITO slides and analyzed the cellular lipid extract samples with the nanoESI-MS under identical setting. Briefly, two ITO slides were seeded with high-density of PANC1 cells (ca. 2×10^4^ cells), the ITO slides were harvested after overnight cell adherence. One cell slide sample was treated with the same protocol as used in SC-MSI, which included PBS washes, fixation for 15 min with chilled 4% formaldehyde in PBS, and 50 mM ammonium acetate aqueous washes. Another cell slide sample was washed with PBS and ammonium acetate, but without formaldehyde fixation. Then, the cell slides were dried under vacuum. After drying the cell slides under vacuum, lipid extraction was performed using 0.5 mL 50% ACN/MeOH solution. The cellular lipid extracts were diluted 100 times with MeOH and subjected to nanoESI-MS analysis in both positive and negative mode in the Orbitrap MS platform. The result is shown in **Supplementary Figure 26**, showing that the lipids signals from the sample treated with 15 min chilled 4% formaldehyde fixing and the control sample are highly consistent in either positive or negative ionization mode. This result also indicates that the washing and fixing pretreatments applied prior to SC-MSI have minimal impact on the lipidome of the cells on the slide. Indeed, it is worth mentioning that caution is required when fixing the single-cell sample with the formaldehyde PBS solution. The fixing time, temperature, and concentration should be set at a low level to avoid excessive fixing, as the excessive fixing can affect the determination of amine-containing lipids such as phosphatidylethanolamine (PE), phosphatidylserine (PS).

**Supplementary Figure 1.** Mass spectra obtained from a single PANC1 cell with different MALDI matrix: (a) DHB and (b) CHCA.

**Supplementary Figure 2.** Single-cell MS imaging result of cell coculture of PSC and PANC1 cells with CHCA matrix deposited on the cells using the TM-sprayer: (a) microscopy images of the cell coculture before the MS imaging, the image is representative of three independent experiments; (b) single-cell MS images of PC (32:0) at *m/z* 734.58 Da. Scale bar is 100 µm.

**Supplementary Figure 3.** Coating the cells with MALDI matrix via sublimation (left, schematic diagram of the home-made sublimation device; right, snapshot of the sublimation device)

**Supplementary Figure 4.** Microscopy optical images of the CHCA matrix deposited on the ITO slide via (a) TM sprayer and (b) dry sublimation, under a magnification of 60× on the microscope. All the images are representative of three independent experiments. The scale bar is 100 *μ*m.

**Supplementary Figure 5.** PSC cells coated with a homogeneous layer of CHCA matrix after the sublimation: (a) microscopy image of the cells before the application of the matrix and (b) microscopy image of the cells after the application of MALDI matrix. The data are representative of three independent experiments. The black lines were drawn on the back side of the ITO slide to annotate the cell region.

**Supplementary Figure 6**. Box plot of signal intensities of lipid standards from 12 regions on the ITO slides: (a) LPC 18:1 ([M+H]^+^, *m/z* 522.3545), (b) PC (18:1-18:1) ([M+H]^+^, *m/z* 786.6007), regions = 12, area of each region is about 160 µm x 160 um, the box plot build based on the signal intensity from each single-spectra (n = 256); (c) overlapped peaks of LPC 18:1 ([M+H]^+^, *m/z* 522.3545) from the average mass spectra of each region (n = 12), (d) overlapped peaks of PC (18:1-18:1) ([M+H]^+^, *m/z* 786.6007) from the average mass spectrum of each region (n = 12), the green shaded rectangular background indicates a mass region of 10 ppm. Among the 12 regions, relative standard deviations (RSDs) for LPC 18:1 ([M+H]^+^, *m/z* 522.3545) were 9.5%–10.5% and the RSDs were 11.8%–14.3% for PC (18:1-18:1) ([M+H]^+^, *m/z* 786.6007), while the average intensities of LPC 18:1 and PC (18:1-18:1) across the 12 regions were quite consistent, with RSDs (n = 12) of 1.35% and 1.67% for LPC 18:1 and PC (18:1-18:1), respectively. All box plots indicate median (center line), 25th and 75th percentiles (bounds of box), and minimum and maximum (whiskers).

**Supplementary Figure 7.** Single-cell MS imaging for PANC1 cells under positive ion mode via sublimation of CHCA matrix: (a) optimal image of the PANC1 cells under microscopy before the MS imaging experiment, the data is representative of three independent experiments. (b-l) MS images of lipids observed from the cells. Scale bar is 200 µm and the mass error tolerance is 10 ppm of each MS image.

**Supplementary Figure 8.** Single-cell MS imaging of SK-N-SH cells under positive ion mode via sublimation of CHCA matrix: (a) optimal image of the SK-N-SH cells took under microscope prior to the MS imaging experiments, the data is representative of three independent experiments. (b-i) MS images of lipids observed from the SK-N-SH cells. Scale bar is 200 µm and the mass error tolerance is 10 ppm of each MS image.

**Supplementary Figure 9. Single-cell MS imaging of the PSC cells:** (a) bright-field image of the PSC cells prior to the SC-MSI, the blue arrow denote the cell nucleus, the data is representative of three independent experiments. MS images of representative lipid species detected from PSC cells (b) Cer (34:1;O2) ([M+H-H_2_O]^+^, *m/z* 520.5044, (c) PC (34:1) ([M + H]^+^, *m/z* 760.5854), (d) PC (36:4) ([M + H]^+^, *m/z* 782.5685), (e) overlay ion images of Cer (34:1;O2) ([M+H-H_2_O]^+^, *m/z* 520.5044, red) and PC (34:1) ([M + H]^+^, *m/z* 760.5854, blue), (f) overlay ion images of Cer (34:1;O2) ([M+H-H_2_O]^+^, *m/z* 520.5044, red) and PC (36:4) ([M + H]^+^, *m/z* 782.5685, green). All SC-MSI images were obtained with mass error tolerance of 5 ppm. Scale bar, 100 µm.

**Supplementary Figure 10.** Average ion mobility heat maps and mass spectra obtained from the PSC cells (a) and PANC1 cells (b) using SC-MALDI-MSI coupled with ion mobility separation under positive ionization mode. The heat map displays a diverse range of ions for each *m/z* value, showing enhanced peak capacity achieved through the integration of the TIMS separation dimension. The mass spectral ranges include *m*/*z* values 700–850 and the heat map incorporates 1/*K*_0_ values 1.35–1.49.

**Supplementary Figure 11.** Representative zoom-in ion mobility heat maps and mass spectra obtained from the PSC cells and PANC1 cells using SC-MALDI-MSI coupled with the ion mobility separation under positive ionization mode. (a) PSC cells at the mass spectral range of *m/z* 762.4–762.8, (b) PANC1 cells at the mass spectrum range of *m/z* range 762.4–762.8, (c) PSC cells at the mass spectral range of *m/z* at 788.4–788.8, and (d) PANC1 cells at the mass spectral range of *m/z* 788.4–788.8.

**Supplementary Figure 12.** Single-cell MS imaging of PSC cells under negative ion mode using DAN as matrix: (a) optical image of the PSC cells under microscopy before the MS imaging experiment, the data is representative of three independent experiments. (b-i) MS images of lipids observed from the cells. Scale bar is 200 µm and the mass error tolerance is 10 ppm of each MS image.

**Supplementary Figure 13**. MALDI-MS/MS of lipid species from single cells: (a) schematic diagram of *in situ* MALDI spotting on individual single cells on the ITO slide, (b-h) representative MS/MS spectra of lipid species from single cells based on MS/MS fragmentation.

**Supplementary Figure 14.** Microscopic images (bright-field) of the PSC cells during the singe-cell MS imaging: (a) PSC cells on the ITO slide prior to the application of MALDI matrix, (b) The same area of PSC cells after the singe-cell MS imaging (region ① is the MS imaging measurement area and region ② is the area without laser irradiation), (c) Microscopic images of the PSC cells on the same area after singe-cell MS imaging with the remaining MALDI matrix washed away using 50 mM ammonium acetate aqueous solution. All the data are representative of three independent experiments.

**Supplementary Figure 15. Multimode SC-MSI of individual cells:** (a) mass spectra of PSC cells obtained from the first imaging run in positive mode with CHCA matrix, (b) mass spectra of the same PSC cell obtained in a follow-up MS imaging acquisition in negative mode with DAN matrix.

**Supplementary Figure 16. Mass spectra obtained from PSC SC samples**: (a) negative ionization mode mass spectra obtained from PSC cells undergo single-polarity ionization, (b) negative ionization mode mass spectra obtained from PSC cells undergo dual-polarity ionization (1^st^ positive MSI using the CHCA matrix, followed by negative MSI with DAN matrix), (c) zoom-in mass spectra of spectrum a, (d) zoom-in mass spectra of spectrum b. Here we compared the negative mode mass spectra obtained from PSC cells that without prior-positive-MSI and the mass spectra of PSC cells with dual-polarity ionization (positive MSI using the CHCA matrix followed by negative MSI with DAN matrix).

**Supplementary Figure 17**. **Sequential MS imaging of the same single cells using the dual-ionization strategy:** (a) bright-field image of the PSC cells on the ITO slide prior to MS imaging, the data is representative of three independent experiments; (b) bright-field image of the PSC cells after the positive MS imaging (the cells were coated with CHCA matrix and the dash rectangle indicated the MSI area), the data is representative of three independent experiments; (c) microscope optical image of the PSC cells after the CHCA matrix being washed away using 50 mM ammonium acetate, the data is representative of three independent experiments; (d)-(g) representative MS images of the PSC cells obtained via the positive mode ionization SC-MSI, (h)-(k) representative MS images of the same PSC cells obtained from the subsequent negative mode ionization SC-MSI. All SC-MSI images were obtained with mass error tolerance of 10 ppm. Scale bar, 400 µm.

**Supplementary Figure 18. UMAP analysis of mass spectrometry fingerprints from the PSC cells (n=68).**

**Supplementary Figure 19. PCA and UMAP analysis of the PSC cell MSI data shown in Supplementary Figure 17:** (a) PCA result of the PSC cells imaged under positive mode acquisition, (b) PCA result of the PSC cells imaged under negative mode acquisition, (c) PCA result of the PSC cells with positive and negative data combined for each cell accordingly, (d) UMAP result of the PSC cells imaged under positive mode acquisition, (e) UMAP result of the PSC cells imaged under negative mode acquisition, (f) UMAP result of the PSC cells with positive and negative ion mode data combined for each cell accordingly. Each dot represents a PSC cell. For the PCA and UMAP analysis, briefly, the overall average mass spectra from 22 PSC cells were extracted from both the positive mode MSI and negative mode MSI data sets. Under the condition of no data intensity filtering of the raw mass spectra, for each single cell, 29890 m/z features were extracted from the positive MSI data, and 21563 m/z features were extracted from the negative MSI data for the analysis. After a combination of the positive and negative ion mode mass spectra for each cell accordingly, a total of 51453 m/z features from each single cell were used for the analysis.

**Supplementary Figure 20. PCA and UMAP analysis of the MSI data from the PSC and PANC1 cells:** (a) PCA result based on the mass spectra, (b) UMAP result based on the mass spectra data, (c) PCA result based on the mass spectra and CCS information, (d) UMAP result based on the mass spectra and CCS information. Here, each color-dot represents a single cell, a total of 37 PSC cells and 43 PANC1 cells were included in the analysis. The successful separation of PSC and PANC1 cells was demonstrated in the PCA and UMAP analysis. Upon comparing the results obtained from the analysis using only the mass spectra data (a and b) to those incorporating the CCS information (c and d), it was observed that the inclusion of CCS data led to an improved performance in grouping the cells within the PCA and UMAP results.

**Supplementary Figure 21.** Both PANC1 and PSC cells were characterized based on their cell surface markers and protein expression by collaborators before use, these two cell types were from different lineage and have different morphologies. (Top) Bright field images of PANC1, PSC and PANC1+PSC in coculture. All data are representative of three independent experiments. All scale bars were equal to 50 μm. (Bottom) NIH ImageJ software was used to measure cell length, width, areas and perimeters and derived length to width ratio and circularity when PANC1 and PSC cultured individually. The length to width ratio was roughly 1.2:1 for PANC1 whereas that ratio was 2.6:1 in PSC. We did not find these parameters change when these two types of cells cultured together in coculture system. These cells have different shapes and sizes on the ITO slide that can be easily differentiated by morphology. PANC1 cells are typically round with a diameter of 18–25 μm, resembling typical cancer epithelial cells. On the other hand, PSC cells are elongated with protrusions, having larger length to width ratios. The average size of PSC is 114 × 43 μm², which is typically 2.8 times larger than that of PANC1. Two tailed t-test with two samples assuming unequal variances, significant difference was determined by a two-tailed Student’s *t*-test, n = 30 (**p < 0.001), all box plots indicate median (center line), 25th and 75th percentiles (bounds of box), and minimum and maximum (whiskers).

**Supplementary Figure 22. MALDI** **Mass spectra obtained from single-cell sample under positive mode:** (a) PANC1 cell and (b) PSC cell.

**Supplementary Figure 23. Analysis of the lipid species from PANC1 and PSC cells:** (a) volcano plot of lipid species variations between the PANC1 and PSC cells and (b) classification performance of the machine learning models of support vector machine (SVM), random forest classifier (RF), and multilayer perceptron (MLP).

**Supplementary Figure 24. NanoESI-MS analysis lipid standards mixture samples**: (a) and (c) are mass spectra of control sample obtained under positive and negative ionization mode, respectively; (b) and (d) are mass spectra of the washing treated sample obtained from positive and negative ionization mode, respectively. The LPC 18:1, PC 18:1-18:1, PC 18:0-18:1 lipid species were detected as protonated ions and the FA 16:1, FA 18:1, PS 18:0-18:1, PE 18:0-18:1 were detected as deprotonated ions in the spectra. The results indicated that the washing and fixing procedure used in the single-cell MSI sample pretreatments have minimal impact on the lipid detection and cell lipidome.

**Supplementary Figure 25. NanoESI-MS analysis of cellular lipid extracts:** (a) positive ion mode mass spectrum of cellular lipid extract from PANC1 cell slides without fixation, (b) positive ion mode mass spectrum of cellular lipid extract from PANC1 cell slides with 15 min chilled 4% formaldehyde fixing, (c) negative ion mode spectra of cellular lipid extract from PANC1 cell slides without fixing, (d) negative ion mode mass spectrum of cellular lipid extract from PANC1 cell slides with 15 min chilled 4% formaldehyde fixing.

**Supplementary Figure 26.** SC-MSI requires high mass resolution to avoid the isobaric overlaps among different lipid species and careful consideration when construct the MS images of SC, using the isotopic pattern of PC(32:2) (a) and PC(32:1) (b) as an example: theoretically, the accurate *m/z* of [M+2] isotopologue for PC(32:2) ([M + H]^+^) is 732.54451 Da with a relative abundance of 11.45% corresponding to its monoisotopic mass; while the monoisotopic mass for PC(32:1) ([M + H]^+^) is 732.55378 Da. There is a 13 ppm mass difference between the [M+2] isotopologue of PC(32:2) (m/z 732.54451) and the PC(32:1) monoisotopic (*m/z* 732.55378).

**Supplementary Figure 27.** H&E-stained histological image of the mouse cerebellum tissue section, the bottom panel shows the zoom-in image of the outlined region from the top panel. The H&E-stained histological image of brain tissue section showed that the nuclei size of brain cells is usually over 5 µm, while the brain cells are surrounded by extracellular matrix. All data are representative of three independent experiments.

**Supplementary Figure 28.** The spatial distribution of representative lipids across a sagittal mouse brain tissue section obtained by MSI under 10 µm lateral resolution.

**Supplementary Figure 29. MS imaging of mouse brain tissue at 10 µm spatial resolution:** (a) MSI image of PC(32:0) ([M+H]^+^, *m/z* 734.5666) from a sagittal mouse brain tissue section, (b) magnification H&E histological image of the extracellular region as highlighted by the white rectangle in (a) showing subregions in the cerebellum (outlines with white dished lines) including Molecular layer (denoted by the green arrow), Granular layer (denoted by the red arrow), Fiber tracts (denoted by the blue arrow), and Fastigial nucleus (denoted by the yellow arrow), (c) high-magnification MSI image of PC (32:0) ([M+H]^+^, *m/z* 734.5666), (d) PC (34:1) ([M + H]^+^, *m/z* 760.5833), (e) PC (36:1) ([M+H]^+^, *m/z* 788.6129), the hollow arrows denotes higher signal intensity of PC (36:1) detected, (f) PC (38:6) ([M+H]^+^, *m/z* 860.5658), (g) PC (40:6) ([M+H]^+^, *m/z* 834.5982). All MSI images were obtained with mass error tolerance of 10 ppm. Interestingly, layer-specific regions (ca. 30-40 µm of thickness) from the boundaries of Molecular layer and Granular layer as well as Granular layer and Fiber tracts had high signal intensity of PC (36:1) at *m/z* 788.6129, whereas low signal intensity of the PC (36:1) was found in the inner regions of the Molecular layer, Granular layer, and Fiber tracts (**e**). These results indicate that cell layer-specific lipidome diversity can be revealed even in the subregion of brain cerebellum with the assistance of high spatial resolution MSI.

**Supplementary Figure 30.** A custom-developed graphical user interface (GUI), termed as MSI Parser, provides a user-friendly platform for analyzing single-cell MSI data: (a) snapshot of the MSI Parser interface, (b) workflow for the single-cell MS imaging data analysis. This program, code, and its accompanying documentation are open-source and available at <https://github.com/lingjunli-research/Automatic-MSI-Spectra-Extraction>.

**Supplementary Figure 31.** Single-cell ROI picking of PANC1 cell MSI data using the SCiLS Lab manually and the MSI Parser automated workflow: (a) microscopic brightfield image of the PANC1 cell prior to the MS imaging, the data is representative of three independent experiments; (b) ion image of PC (34:1) (*m/z* 760.58) from the PANC1 cells; (c) single-cell ROIs picked using the SCiLS Lab manually, in which 87 PANC1 cells were tentatively picked based on comparison of the optical image and the ion images. Here, each circle presents a single cell region, where some of PANC1 cells may be missed during the manual ROI picking process; (d) single-cell ROIs picked using the proposed MSI Parser automated workflow, in which 198 cells were picked, where each color-dot represents a single cell region. The results obtained from the automated workflow successfully included all of the manually-picked ROIs. The implementation of the automated workflow has considerably improved the efficiency of data processing, with which the ROI picking can be completed within minutes, whereas the manual picking via SCiLS Lab would typically take several hours.

**Supplementary Figure 32.** Output results from automated workflow for the analysis of MSI data from PANC1 and PSC cells: (a) PCA analysis result, (b) UMAP analysis result, (c) TSNE analysis result, (d) machine learning confusion matrix displays the result of the testing data using the Random Forest model. In the PCA, UMAP, and TSNE results, one dot presents a single cell. MSI data from PANC1 and PSC cells were imported in the MSI Parser automated workflow separately, in which 48 PSC cells and 198 PANC1 cells were extracted from the PSC and PANC1 MSI data, respectively. These results demonstrate that the PSC and PANC1 cells could be successfully distinguished under the statistical analysis based on the automated workflow.

**Supplementary Table 1.** Details on machine learning models trained and validated for supervised classification on SC data.

| Model | Optimized hyperparameters | Performance |
| --- | --- | --- |
| Support vector machine | Regularization parameter: 1.0  Kernel function: rbf | Accuracy: 100%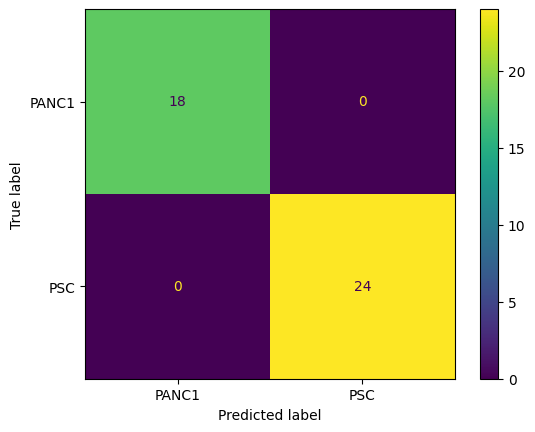 |
| Random forest classifier | n_estimators: 100  Criterion: gini  Maximum depth: none | Accuracy: 100%  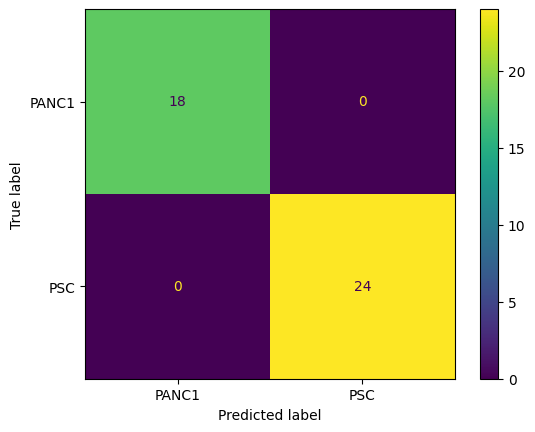 |
| Multilayer perceptron | hidden_layer_sizes: 100  Activation: relu  Solver: adam  Alpha: 0.0001  Batch size: auto  Learning rate: 0.001 | Accuracy: 100%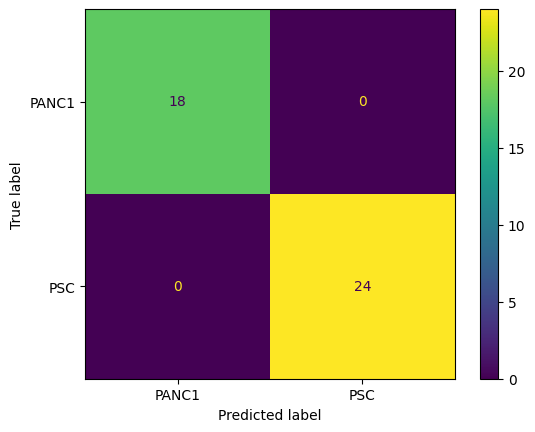 |

**Supplementary References**

1. Leger, S.; Black, W. C.; Deschenes, D.; Dolman, S.; Falgueyret, J. P.; Gagnon, M.; Guiral, S.; Huang, Z.; Guay, J.; Leblanc, Y.; Li, C. S.; Masse, F.; Oballa, R.; Zhang, L., Synthesis and biological activity of a potent and orally bioavailable SCD inhibitor (MF-438). *Bioorganic & Medicinal Chemistry Letters* **2010,** *20* (2), 499-502.
