# Supplementary material for: Single-cell lipidomics enabled by dual-polarity ionization and ion mobility-mass spectrometry imaging
Source: Nat Commun. 2023 Aug 25;14:5185. doi: 10.1038/s41467-023-40512-6 (PMC10457347; doi:10.1038/s41467-023-40512-6)
Supplement: Supplementary file 3 — Description of additional supplementary files [file 41467_2023_40512_MOESM3_ESM.pdf]

Description of Additional Supplementary Files

Supplementary Data 1

Description: List of the identified lipid species from single cell samples using SC-MSI under positive ion mode.

Supplementary Data 2

Description: List of the identified lipid species from single cell samples using SC-MSI under negative ion mode.

Supplementary Data 3

Description: List of lipids exhibiting significant difference between PANC1 and PSC cells from the co-culture sample.

Supplementary Data 4

Description: Lipidomic search results from cell samples and mouse brain samples.
