# Supplementary material for: Single-cell lipidomics enabled by dual-polarity ionization and ion mobility-mass spectrometry imaging
Source: Nat Commun. 2023 Aug 25;14:5185. doi: 10.1038/s41467-023-40512-6 (PMC10457347; doi:10.1038/s41467-023-40512-6)
Supplement: Supplementary file 8 — Reporting Summary [file 41467_2023_40512_MOESM8_ESM.pdf]

Corresponding author(s): Lingjun Li
Last updated by author(s): July 9, 2023

Reporting Summary

Nature Portfolio wishes to improve the reproducibility of the work that we publish. This form provides structure for consistency and transparency in reporting. For further information on Nature Portfolio policies, see our Editorial Policies and the Editorial Policy Checklist.

Statistics

For all statistical analyses, confirm that the following items are present in the figure legend, table legend, main text, or Methods section.

n/a	Confirmed	
☐	☒	The exact sample size (*n*) for each experimental group/condition, given as a discrete number and unit of measurement
☐	☒	A statement on whether measurements were taken from distinct samples or whether the same sample was measured repeatedly
☐	☒	The statistical test(s) used AND whether they are one- or two-sided<br>*Only common tests should be described solely by name; describe more complex techniques in the Methods section.*
☐	☒	A description of all covariates tested
☐	☒	A description of any assumptions or corrections, such as tests of normality and adjustment for multiple comparisons
☐	☒	A full description of the statistical parameters including central tendency (e.g. means) or other basic estimates (e.g. regression coefficient) AND variation (e.g. standard deviation) or associated estimates of uncertainty (e.g. confidence intervals)
☐	☒	For null hypothesis testing, the test statistic (e.g. *F*, *t*, *r*) with confidence intervals, effect sizes, degrees of freedom and *P* value noted<br>*Give P values as exact values whenever suitable.*
☒	☐	For Bayesian analysis, information on the choice of priors and Markov chain Monte Carlo settings
☒	☐	For hierarchical and complex designs, identification of the appropriate level for tests and full reporting of outcomes
☐	☒	Estimates of effect sizes (e.g. Cohen's *d*, Pearson's *r*), indicating how they were calculated

Our web collection on statistics for biologists contains articles on many of the points above.

Software and code

Policy information about availability of computer code

Data collection	For MS imaging: flexImaging 7.2, TimsControl 4.1, SCiLS Lab 2023c Pro;<br>For LC-MS/MS acquisition: Thermo Scientific Xcalibur 4.2 with Foundation 3.1 SP5, Orbitrap Fusion Lumos Tune Application 3.1, Thermo Scientific SII for Xcalibur 1.4, Bruker otofControl Version 6.2.
Data analysis	The cell lipidomics data was analyzed by MSDIAL (Version 4.80), MetaboScape version 8.0.1, ImageJ version 1.41., Python 3.10., Compass DataAnalysis Version 6.1.<br>Custom codes is available at https://github.com/lingjunli-research/Automatic-MSI-Spectra-Extraction with doi: 10.5281/zenodo.8097359.

For manuscripts utilizing custom algorithms or software that are central to the research but not yet described in published literature, software must be made available to editors and reviewers. We strongly encourage code deposition in a community repository (e.g. GitHub). See the Nature Portfolio guidelines for submitting code & software for further information.

Data

Policy information about availability of data

All manuscripts must include a data availability statement. This statement should provide the following information, where applicable:

- Accession codes, unique identifiers, or web links for publicly available datasets
- A description of any restrictions on data availability
- For clinical datasets or third party data, please ensure that the statement adheres to our policy

Data searching used including LIPID MAPS database (https://www.lipidmaps.org/) and Human Metabolome Database (https://hmdb.ca/).
The data that support the findings of this study are available in the supplementary material of this article. All data are available from the authors upon reasonable request.

Human research participants

Policy information about studies involving human research participants and Sex and Gender in Research.

Reporting on sex and gender	*Use the terms sex (biological attribute) and gender (shaped by social and cultural circumstances) carefully in order to avoid confusing both terms. Indicate if findings apply to only one sex or gender; describe whether sex and gender were considered in study design whether sex and/or gender was determined based on self-reporting or assigned and methods used. Provide in the source data disaggregated sex and gender data where this information has been collected, and consent has been obtained for sharing of individual-level data; provide overall numbers in this Reporting Summary. Please state if this information has not been collected. Report sex- and gender-based analyses where performed, justify reasons for lack of sex- and gender-based analysis.*
Population characteristics	*Describe the covariate-relevant population characteristics of the human research participants (e.g. age, genotypic information, past and current diagnosis and treatment categories). If you filled out the behavioural & social sciences study design questions and have nothing to add here, write "See above."*
Recruitment	*Describe how participants were recruited. Outline any potential self-selection bias or other biases that may be present and how these are likely to impact results.*
Ethics oversight	*Identify the organization(s) that approved the study protocol.*

Note that full information on the approval of the study protocol must also be provided in the manuscript.

Field-specific reporting

Please select the one below that is the best fit for your research. If you are not sure, read the appropriate sections before making your selection.

☒ Life sciences ☐ Behavioural & social sciences ☐ Ecological, evolutionary & environmental sciences

For a reference copy of the document with all sections, see nature.com/documents/nr-reporting-summary-flat.pdf

Life sciences study design

All studies must disclose on these points even when the disclosure is negative.

Sample size	The sample size was chosen based on previous experience, considering the number of cells necessary to accurately identify significant biological differences. All experiments were performed at least three biological replicates. The exact sample size for each experiments is reported in the relevant figure legend. Multiple tests and analyses were performed as described in the manuscript to ensure that the samples are representative and the results are conclusive.
Data exclusions	No data was excluded from the analyses.
Replication	The number of replicates for each specific experiment is indicated throughout the manuscript text, figure legends and methods. All attempts of replication were successful.
Randomization	Samples were separated into groups based on the cell type and treatments. All samples were processed in parallel.
Blinding	There was no blinding in this study because intervention bias was not an issue given the nature of the study. Statistical analyses performed following mass spectrometry are inherently unbiased approaches.

Reporting for specific materials, systems and methods

We require information from authors about some types of materials, experimental systems and methods used in many studies. Here, indicate whether each material, system or method listed is relevant to your study. If you are not sure if a list item applies to your research, read the appropriate section before selecting a response.

Materials & experimental systems

n/a	Involved in the study
☒	☐ Antibodies
☐	☒ Eukaryotic cell lines
☒	☐ Palaeontology and archaeology
☐	☒ Animals and other organisms
☒	☐ Clinical data
☒	☐ Dual use research of concern

Methods

n/a	Involved in the study
☒	☐ ChIP-seq
☒	☐ Flow cytometry
☒	☐ MRI-based neuroimaging

Eukaryotic cell lines

Policy information about cell lines and Sex and Gender in Research

Cell line source(s)	Pancreatic cancer cell line PANC-1 and neuroblastoma cell line (SK-N-SH) were purchased from American Type Culture Collection. Human primary pancreatic stellate cells (PSCs) were isolated from PDAC tumor specimen resected from patients at the University of Wisconsin Carbone Cancer Center (UWCCC) with informed consent.
Authentication	Cell were authenticated at their source (e.g., ATCC). No extra authentication was applied in the study.
Mycoplasma contamination	The cell line was not tested for mycoplasma contamination.
Commonly misidentified lines (See ICLAC register)	No commonly misidentified cell lines were used.

Animals and other research organisms

Policy information about studies involving animals; ARRIVE guidelines recommended for reporting animal research, and Sex and Gender in Research

Laboratory animals	Female Wild Type mice, age of 40 weeks. Mice were housed in facilities with a standard light-dark cycle, humidity of 50% at 24 °C.
Wild animals	This study did not involve wild animals.
Reporting on sex	The sex- and gender-based analysis is not relevant to the study.
Field-collected samples	This study did not involve samples collected from the field.
Ethics oversight	Animal care and experimental procedures were performed with approval from the Institutional Animal Care and Use Committees from the University of Wisconsin-Madison and William S. Middleton Memorial Veterans Affairs

Note that full information on the approval of the study protocol must also be provided in the manuscript.
